# C-852-06. Minutes to Hours: Continuous Calorimetry Reveals Dynamic Energy Needs in Severe Burns a Case Series

**DOI:** 10.1093/jbcr/irag033.066

**Published:** 2026-04-06

**Authors:** Matthew Curtis, Sheela S Thomas, Nicole P Bernal, John H Loftus, Beth McGuire

**Affiliations:** The Ohio State University Wexner Medical Center, Columbus, OH; The Ohio State University Wexner Medical Center, Columbus, OH; The Ohio State University Comprehensive Burn Center, Columbus, OH; The Ohio State University Comprehensive Burn Center, Columbus, OH; The Ohio State University Comprehensive Burn Center, Columbus, OH

## Abstract

**Introduction:**

Severe burns provoke a profound hypermetabolic surge, dramatically increasing energy demands and accelerating protein and muscle loss. Accurate resting energy expenditure (REE) is essential for tailored nutrition, but short indirect calorimetry (IC) measurements often miss critical daily fluctuations. Extended monitor-based indirect calorimetry more accurately reflects dynamic resting energy expenditure in severely burned patients than brief ventilator-derived IC or predictive equations, due to its ability to capture patient-specific metabolic fluctuations.

**Methods:**

We evaluated continuous monitor-based IC REE against brief ventilator-derived IC readings and Milner equation estimates. All patients underwent repeated paired REE studies during the first 22 days of admission. Each study included: (1) a continuous monitor-based calorimetry (6–12 h), (2) a 10-min ventilator REE (CV < 5%), and (3) a Milner equation estimate. Descriptive statistics of estimated and measured energy expenditure are presented as means ± standard deviation and ranges.

**Results:**

Three male burn patients (ages 20, 21, and 48) with TBSA of 50%, 73%, and 50% and admission weights of 116, 103, and 106 kg were monitored. Monitor-derived IC REE was 18% and 17% higher than ventilator-derived values for subjects 1 and 2, and 3% lower for subject 3. Compared to Milner estimates, monitor-derived REE was 5% and 10% lower for subjects 1 and 3, and 4% higher for subject 2.

**Conclusions:**

Monitor-based IC REE is a novel, feasible, and clinically valuable method for assessing energy needs in critically ill burn patients, offering a dynamic, individualized approach. While ventilator-derived IC REE showed low variability, it differed notably from monitor-based values. The Milner equation aligned closely (within 10%) across patients, likely due to its incorporation of key clinical factors. Subject 2, although not the largest burn showed larger discrepancies between IC methods but maintained consistency with Milner estimates. These findings highlight the limitations of static methods and support further research into dynamic, patient-centered nutritional strategies.

**Applicability of Research to Practice:**

Continuous monitor-derived IC is a novel, feasible and clinically valuable approach for assessing dynamic energy needs in critically ill burn populations, capturing fluctuations missed by short ventilator-derived readings.

**Funding for the Study:**

N/A.

